# Supplementary material for: Detection of gene communities in multi-networks reveals cancer drivers
Source: Sci Rep. 2015 Dec 7;5:17386. doi: 10.1038/srep17386 (PMC4671005; doi:10.1038/srep17386)
Supplement: Supplementary Information [file srep17386-s1.pdf]

Detection of gene communities in multi-networks reveals cancer drivers

Laura Cantini[1,2] *, Enzo Medico[1,3]*, Santo Fortunato[4] and Michele Caselle[5]

[1]Università di Torino, Department of Oncology, Candiolo, Italy

[2]Politecnico di Torino, Department of Control and Computer Engineering, Torino, Italy

[3]Candiolo Cancer Institute, FPO IRCCS, Candiolo, Italy

[4] Department of Computer Science, Aalto University School of Science, Aalto, Finland

[5]Università di Torino, Department of Physics and INFN, Torino, Italy

*Corresponding author:

Laura Cantini, Politecnico di Torino, Department of Control and Computer Engineering, Cso Duca degli Abruzzi 24, 10129 Torino, Italy, Tel. +39-011-0907072 email: lcantini@unito.it

Enzo Medico, Università degli Studi di Torino, S.P. 142, km 3,95 - 10060 Candiolo (TO), Italy

Tel. +39-011-9933234 Fax: +39-011-9933225 email: enzo.medico@unito.it

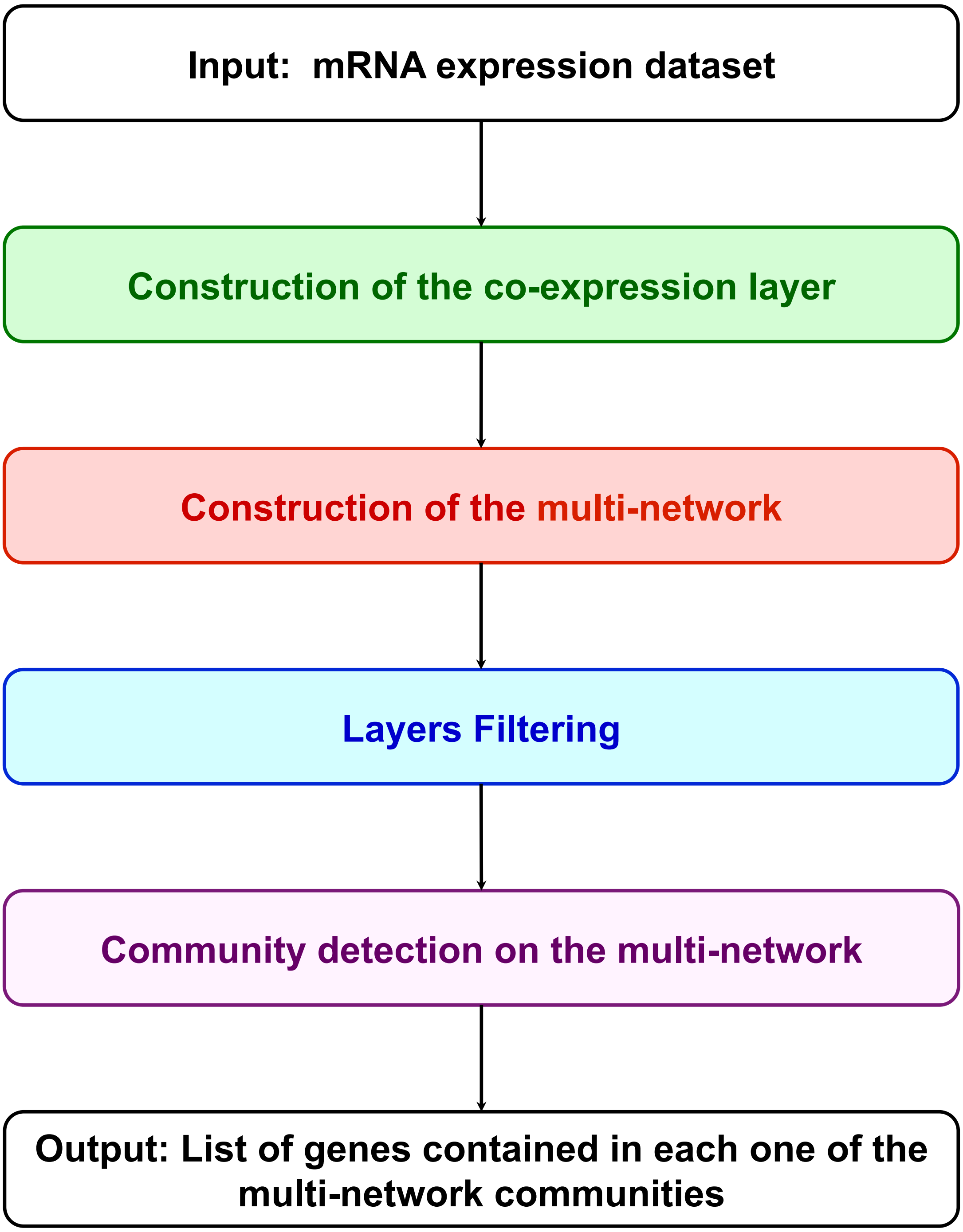

Supplementary Figure S1: Schematic representation of the procedure proposed in the paper.

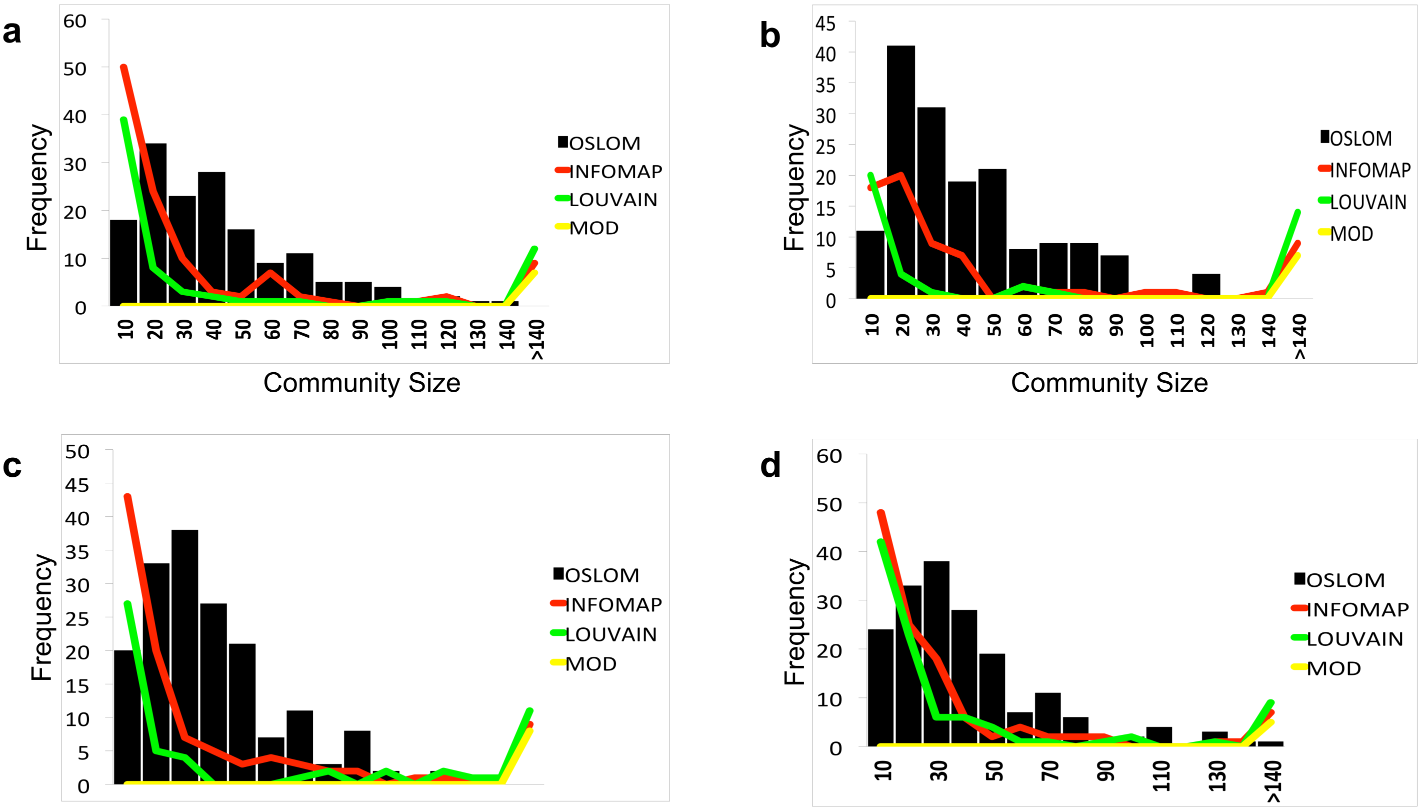

Supplementary Figure S2: Comparison of the four community detection algorithms: size distribution. Histograms reporting the size of the communities obtained with OSLOM (black), Infomap (red), Louvain (green), Modularity optimization (yellow) in gastric (a), lung (b), pancreas (c) and colon (d)

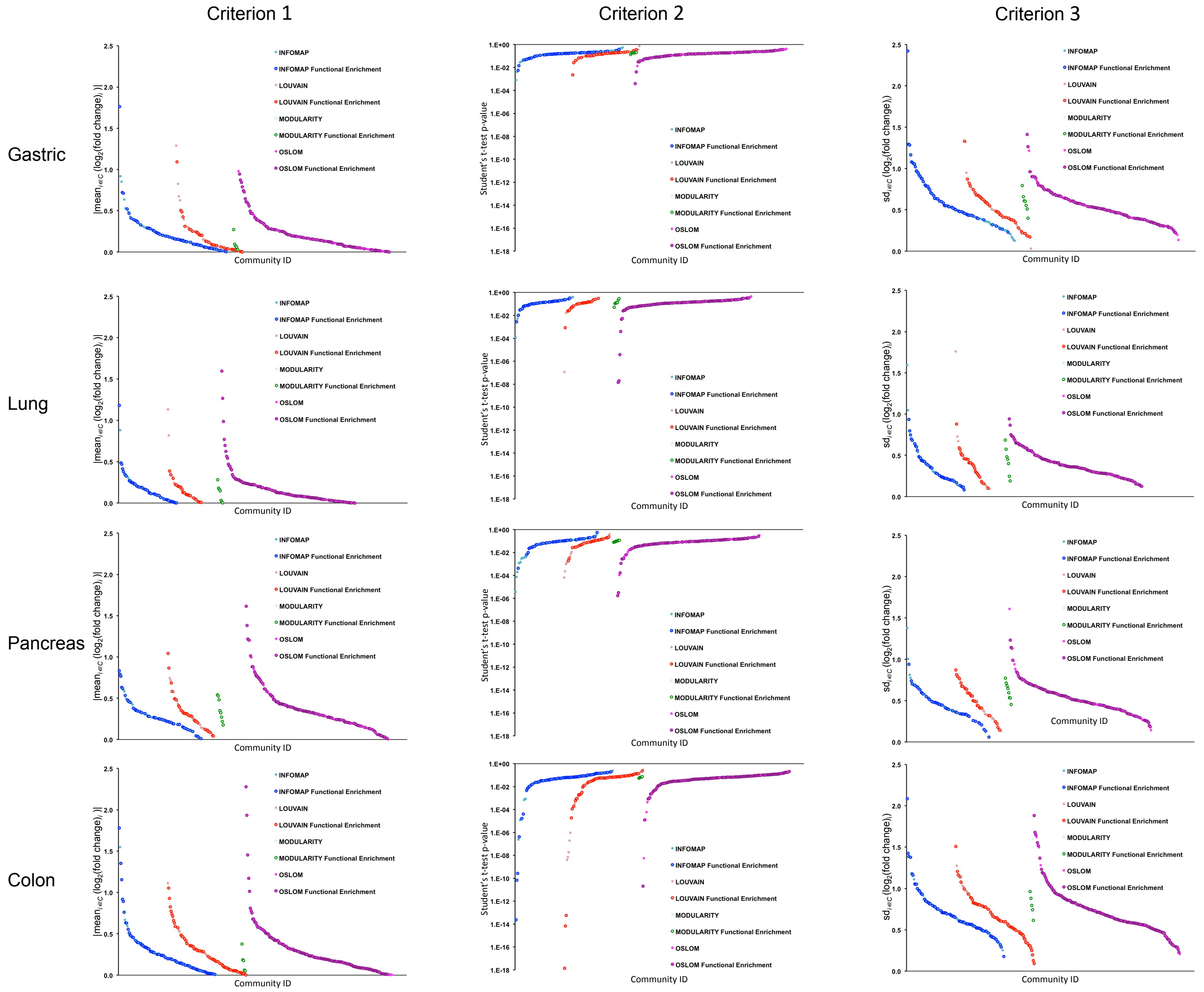

Supplementary Figure S3: Comparison of the four community detection algorithms: differential expression. The four algorithms (Infomap (blue), Louvain (red), Modularity optimization (green) and OSLOM (violet)) were tested in their ability to detect communities differentially expressed between tumor and normal tissues. Each dot in the plot represents a community, a darker colour identifies those communities that are also functionally homogeneous. On the y-axis are reported the results of the three differential expression criteria: $|\text{mean}_{i \in C}\ (\log_2(\text{fold change})_i\ )|$ (a); Student's t-test p-value (b); $\text{sd}_{i \in C}\,(\log_2(\text{fold change})_i)$ (c).

Cantini et al. Supplementary text

Choice of the optimal community detection algorithm **1**
Partition in communities for different values of α **3**
Chromosomal locations and microRNA regulons in Pancreatic cancer **3**
Chromosomal locations 3
microRNA regulons 7
References **8**

Choice of the optimal community detection algorithm

Only four of the five community detection algorithms, discussed in the main text, were considered for this comparison since Label propagation obtained in all the four tissues a partition composed of only one community. For our application to cancer data we evaluated the performances of the four algorithms using two criteria:

- The percentage of functionally homogeneous communities.
- The number of tumor vs normal differentially expressed communities.

For the first criterion the comparison was only made on the tumorous multi-network for simplicity. We first performed an enrichment analysis testing the overlap of the communities with the following categories of annotated gene sets downloaded from MSigDB [1]: positional gene sets, Chemical and Genetic Perturbations (CGP), Canonical Pathways (CP), BioCarta, KEGG gene sets, Reactome gene sets, motif gene sets, GO gene sets. To ensure the specificity of MSigDB terms, we filtered out those general terms associated with > 500 genes. The significance of this overlap was verified through the hypergeometric test, the p-values results of this analysis were then corrected for multiple hypothesis testing according to Benjamini and Hochberg[2]. In this way, for each community, we obtained a list of biological annotations and an associated p-value. To establish which of these p-values were significant, we estimated, for each community, a p-value threshold through a null model. The null model was constructed selecting 1000 times, for each community, a random set of genes of the same dimension of the analyzed community. In each run, the enrichment in biological information of the random set of genes was computed and the minimum p-value was selected. At the end, the distribution of the minimum p-values of all the 1000 runs was studied and the 95th percentile of this distribution was selected as p-value threshold for the studied community.

For the second criterion we used three measures of differential expression. For each of the four tissues, calling T the tumor matrix and N the normal matrix, the measures applied to each multi-network community $\mathcal{C}$, can be written as:

- $|\, mean_{i\in C}(\log_2(fold\ change)_i)| = \overline{|\bar{T}_i - \bar{N}_i\,|}$ where $\bar{T}_i = \sum_{j\epsilon T} \frac{T_{ij}}{|row(T)|}$, $\bar{N}_i = \sum_{j\epsilon N} \frac{N_{ij}}{|row(N)|}$

- Student’s t-test p-value

- $sd_{i\in C}(\log_2(fold\ change)_i) = \sqrt{\frac{(l_i - \bar{l}_i)^2}{|row(T)|}}$, where $l_i = \bar{T}_i - \bar{N}_i$

Each differential expression measure was applied to the multi-network communities identified by the four algorithms and for each measure we identified the best performing algorithm as the one with the maximum value (minimum in the case of the Students's t-test) of the estimator. Then we chose the algorithm with the best performances in the majority of the three tests. The two criteria presented here will be used also in the comparisons discussed in the main text. The algorithm which performed best in all the four tissues, according to both criteria, turned out to be OSLOM. More precisely OSLOM and Modularity optimization were those with the best performances in terms of Biological enrichment. Modularity optimization obtained all biologically enriched communities, but it also identified a really small amount of communities (5-7) compared to those identified by OSLOM (170-190). The results of this analysis are reported in Supplementary Table S14. Instead, with respect to the differential expression analysis, the best performing algorithms were OSLOM in lung and pancreas, Infomap in gastric, while in colon none of the five algorithms performed better than the others in at least two of the tests. A summary of the results for all four tissues are summarized in Supplementary Figure S3. Given the results of the two tests, OSLOM was the algorithm that we chose for our analysis and the communities obtained with this algorithm are reported in Supplementary Tables S3-S6.

Partition in communities for different values of α

For each multi-network community obtained with the optimal α, we selected the community (among those obtained with different values of α) with the highest overlap. To establish which of these overlaps were significant, we estimated, for each community, an overlap threshold through a null model. The null model was constructed selecting 1000 times, for each multi-network community, a random set of genes of the same dimension of the analyzed community. In each run, the overlap of the random set of genes with the multi-network communities was computed and the maximum overlap percentage was selected. At the end, the distribution of the maximal overlaps of all the 1000 runs was studied and the 95th percentile of this distribution was selected as overlap threshold for the studied community. With this choice in the 99% of cases we found a one to one correspondence between communities obtained with different α.

Chromosomal locations and microRNA regulons in Pancreatic cancer

chromosomal locations

As discussed in the paper our analysis may have three further interesting outcomes:

- Out of the hundreds of genes contained in each enriched chromosomal location with our analysis we select only the few which are involved in a common co-regulatory scheme and thus are likely to be the real drivers of the cancer.
- In the communities we find also genes outside the enriched chromosomal locus, related to them non only by a coexpression link but also by regulatory relations and this suggests that they could be part of a common biological pathway which is dysregulated in the tumour.
- In some cases the community is also characterized by a GO or KEGG enriched category which may give some hint to identify the above pathway.

To discuss these points more in detail we considered the Pancreatic multi-network-spefic communities, because they are the ones with the smallest number of enriched chromosomal locations. It is thus a perfect laboratory to test our results since the number is small enough to allow to discuss here all of them. Of note is that we have no false positives in Pancraes: for ALL the eight loci the association with the pancreatic cancer is already well established. In seven cases these are recurrent amplifications, which appear in several tumours and in the pancreatic cancer among the others, while in the remaining case, 6p22 seems to be more specific of the pancreatic cancer and it was identified only recently in two independent genome wide association studies (see below).

Let us discuss these loci more in detail:

- The amplification of 1q21 [3] is one of the most frequent genetic alterations in many solid tumours, including bladder, breast, nasopharyngeal carcinoma, hepatocellular carcinoma,

esophageal tumor, fibrosarcoma of bone, colorectal carcinoma (and accordingly we find it enriched also in the CRC dataset) and in agreement with our finding, also in the pancreatic tumour [4,5]. With 1q32 discussed below is one of the first cancer related chromosomal aberrations reported in the literature [3]. This locus turns out to be enriched in the 106th community of the pancreatic dataset, with a p-value of 10^-3.

- Also 1q32 [3], which shows an enrichment in the 43th community of the Pancreas dataset with a p value of 10^-4, is a common and well studied genetic alteration. It was identified as a specific pancreatic cancer susceptibility locus in a genome-wide association study five years ago [6]. This identification was recently confirmed in [7].
- 6p22 is not a common genetic alteration. It was only recently found associated to pancreatic tumour in two separate studies [8,9]. It seems not to be associated to any other type of tumour, accordingly we found it enriched only in the pancreas dataset. This locus is enriched in the 109th community, with a p-value of 10^-3.
- 11q13 is a chromosomal locus associated to several types of cancer and in particular also to the pancreatic one [5,10]. It is known to be the most common genetic aberration in the adrenocortical carcinoma. Also in this case the locus turns out to be enriched also in the CRC dataset and accordingly it is known to be associated also to the colorectal cancer [11]. This locus is enriched in two communities: the first one, with a p-value of 10^-5 and in the 92nd one, with a p-value of 10^-3.
- 11p15 is a very common genetic alteration in many tumours and was recently found also in pancreatic cancer [12]. This locus is enriched in the 166th community, with p-value 10^-4.
- 17p13 is a very common genetic alteration in many tumors. Its association to pancreatic cancer is rather old [13] and was recently confirmed in [5,8,9]. This locus is enriched in the 23rd community, with a remarkable p-value of 10^-9.
- 17q23 is involved in a recurrent chromosomal amplification in several types of cancer. It was first discovered in breast cancer [14] and then in brain, lung, ovary, bladder, testis, liver and also, in agreement with our findings, in pancreatic tumour [15]. This locus is enriched in the 74th community, with a p-value of 10^-4.
- Finally, also 18p11 is a common genetic alteration, originally found in CRC and more recently also in pancreatic cancers [12]. This locus is enriched in the 11th community, with a p-value of 10^-6.

We now move to the second level of our analysis, with a closer inspection of the gene content of the above communities. We shall discuss in particular, as an example, two cases: the 1q21 and the 11q13 loci.

- The 1q21 locus is enriched in the 106th community which contains 25 genes. Out of them five are located in the 1q21 locus (this explains why we found this locus enriched in this particular community). They are: F11R, HDGF, ILF2, PRCC and VPS72. Among them F11R (also known as JAM-A) was shown a few years ago to be associated with metastasis and poor survival in pancreatic cancer [16]. HDGF is known since 2006 [17] to be a prognostic factor for patients with pancreatic cancer. PRCC is known to be associated to the Papillary Renal Cell Carcinoma (which gives the name to the gene) was recently shown to be mutated also in the pancreatic tumour [18]. Our analysis suggests that the simultaneous presence of these three oncogenes in the same community is not a coincidence and that it is exactly the fact that they are located in the same chromosomal locus which makes alterations of this locus so dangerous. Moreover it is interesting to notice that with our analysis, out of the hundreds of genes contained in this locus we were able to single out three genes with a known important role in the pancreatic tumour. This strongly suggests that also the remaining two could play a role and prioritized their analysis. Indeed VPS72 is involved in two multi-component complexes, the histone acetyltransferase complex TRRAP/TIP60 and the chromatin remodeling SRCAP-containing complex. In particular, the TRRAP/TIP60 complex acetylates nucleosomal histones and is important for transcriptional regulation, double strand DNA break repair and apoptosis. As such it would be not too unlikely to find that it could play a role also in the insurgence of pancreatic cancer. Similarly ILF2 (also known as NF45) is known to have a tumorigenic role in other types of cancer, ranging from CRC [19] to the esophageal squamous cell carcinoma [20]. Again it would be not too unlikely to expect a role also in pancreatic cancer. Notice that, interestingly, both ILF2 [21] and VPS72 play a role, via two independent pathways in DNA damage repair. A simultaneous alteration of their expression levels in pancreatic cancer could reduce the ability of the cell to control DNA aberrations .

- The 11q13 locus is enriched in the first and 92nd communities. Out of the 28 genes belonging to 92nd community, four are contained within the 11q13 locus: FKBP2, RASGRP2, RIN1 and TM7SF2. Remarkably enough the last three of these genes are known to be involved in some type of cancer but none of them was previously associated to the pancreatic cancer. RASGRP2 has been shown to be activated in a mouse model of myeloid leukemia [22]. The expression level of RIN1 has been shown to have a prognostic role both in the gastric adenocarcinoma [23] and in the lung cancer [24]. Similarly, also TM7SF2 has been shown to have a prognostic role in the adrenocortical carcinoma [25]. This agrees with the

remark we made above on the fact that aberrations in this particular locus are strongly correlated with the adrenocortical carcinoma. Our analysis supports a role for these genes also in the pancreatic cancer, maybe within the same pathways already observed for other types of cancer. It would be interesting to test this conjecture. However the most interesting case is probably that of the fourth gene: FKBP2. FKBP2 belongs to the family of FKBP proteins which are highly expressed in the cell and show a high degree of conservation across species. They modulate several signal transduction pathways in the cell and in the last few years they have been shown to play an important role in cancer related pathways. (for a recent review see [26]). In particular, it has been recently shown that variability in the expression level of another protein of this family: FKBP5 is associated to the variation in response to various chemotherapeutic agents in pancreatic cancer and in particular for gemcitabine [27], a first line treatment for pancreatic cancer. A similar involvement in pancreatic cancer for FKBP2 was never observed up to now, however our analysis strongly supports this possibility and suggests that it could be worthwhile to explore this research line.

Finally, as an example of the third level of analysis let us discuss the locus 1q32:

- 1q32 is enriched in the 43rd community. This community contains 45 genes. Out of them five belong to the locus: ATF3, BTG2, CD46, IRF6 and PPP1R15B. As in the previous cases, also for this locus three out of these five genes ATF3 BTG2 and CD46 are already known markers of pancreatic cancer. ATF3 is a well known oncosuppressor both in pancreatic and in other types of cancer [28]. also BTG2 is an oncosuppressor whose relevance in other types of cancer is well known while its role in pancreatic cancer has been proved only recently [29]. Instead, CD46 in pancreatic cancer has the opposite role. It is a cell-surface glycoprotein involved in protection of tumour cells against complement-mediated cytotoxicity and its activation is controlled by the oncogene STAT3 [30]. What is more interesting for our purposes is that in this case we have some more information on the possible pathways in which these genes, and the other belonging to the community, are involved. Looking at the enrichment analysis for this community we find a few functional categories with rather good p-values. In particular we find the so called DREAM pathway which involves the JUN and FOS regulators. Indeed looking at the other genes belonging to the community we find several genes of the JUN and FOS families, with an over-representation which is clearly statistically significant. Moreover a closer inspection to the

gene set allows to find some already known synergistic interactions among these genes. In particular ATF3 and BTG2 are both involved in the pathways which allow p53 to exert its oncosuppressor function [31]. They are key players in two alternative pathways and thus their simultaneous alteration could have dramatic consequences. As mentioned above CD46 is regulated by STAT3 [30] which is known to act synergistically with JUN and FOS. Finally it was shown a few years ago that ATF3 is induced in pancreatic cancer by one of the other genes in the community: NR4A1 [32]. All these findings point to a cooperative role of several genes of the community (not only those belonging to the selected locus but also the other) in the **apoptotic** process and more generally in **cell survival.** This intuition is supported by the results obtained on community 43 through the Ingenuity Pathway Analysis software (IPA). In fact, we applied IPA to the log2fold change of expression between tumor and normal tissue of the genes constituting community 43 and we considered the Diseases or Functions Annotation, the results are reported in Supplementary Table S15. As shown in that table, the 43rd community is significantly enriched in genes annotated to be involved in the regulation of cell death and apoptosis, in particular according to the IPA analysis these functions result to be decreased in tumor in respect to normal tissue. Moreover the following community is enriched in STAT3 targets (p-value 3.02E-6), that is activated according to IPA analysis with an activation z-score of 2.714.

miRNA regulons

Among the microRNAs significant in at least one community in Pancreas, miR-383 is known to control apoptosis in cancer through the regulation of GADD45G [33], which is one of the genes contained in the 3rd community in which the microRNA targets were enriched. MiR-33a inhibits tumor cell proliferation [34,35], moreover it might function as a tumor suppressor, targeting the 3'UTR of β-catenin and affecting cell growth, apoptosis, EMT and GEM resistance [35]. MiR-337 was found to be associated with longer survival in pancreatic cancer [36]. In particular, it targets HOXB7 causing a significant suppression of PDAC cell proliferation and invasion [37]. MiR-302c is part of the miR-302 cluster whose target genes are known to be involved in developmental signaling. In human, miR-302 cluster is highly expressed in hESCs and iPSCs, and plays a critical role in regulating cell stemness and pluripotency [38]. MiR-153 inhibits PDAC cell migration and invasion by targeting SNAI1, its expression is also an independent prognostic marker for predicting 3-year survival of pancreatic ductal adenocarcinoma (PDAC) patients [39]. The role of mir-153 in tumorogenesis was highlighted also through a bioinformatics analysis in[40]. MiR-365 directly targets apoptosis-mediating molecules, SHC1 and BAX, in pancreatic cancer cells [41]. MiR-183 is an EMT inhibitor

and favors epithelial differentiation, in many tumors [42] and also in pancreas [43,44]. Moreover it was found to be aberrantly expressed during pancreatic carcinogenesis [45]. MiR-373 is down-regulated in pancreatic cancer, and its re-expression represses the invasiveness of pancreatic cancer cells [46]. Moreover it is involved in ZIP4-CREB-miR-373 signaling axis that promotes pancreatic cancer growth, through silencing on key tumour suppressor molecules including TP53INP1, LATS2 and CD44 [47].

REFERENCES

1. Subramanian, A. *et al.* Gene set enrichment analysis: a knowledge-based approach for interpreting genome-wide expression profiles. *Proc Natl Acad Sci U S A* **102**, 15545-50 (2005).
2. Benjamini, Y., Drai, D., Elmer, G., Kafkafi, N. & Golani, I. Controlling the false discovery rate in behavior genetics research. *Behav Brain Res* **125**, 279-84 (2001).
3. Kakati, S., Song, S.Y. & Sandberg, A.A. Chromosomes and causation of human cancer and leukemia. XXII. Karyotypic changes in malignant melanoma. *Cancer* **40**, 1173-81 (1977).
4. Chen, Y.J., Vortmeyer, A., Zhuang, Z., Huang, S. & Jensen, R.T. Loss of heterozygosity of chromosome 1q in gastrinomas: occurrence and prognostic significance. *Cancer Res* **63**, 817-23 (2003).
5. Gutiérrez, M.L. *et al.* Association between genetic subgroups of pancreatic ductal adenocarcinoma defined by high density 500 K SNP-arrays and tumor histopathology. *PLoS One* **6**, e22315 (2011).
6. Petersen, G.M. *et al.* A genome-wide association study identifies pancreatic cancer susceptibility loci on chromosomes 13q22.1, 1q32.1 and 5p15.33. *Nat Genet* **42**, 224-8 (2010).
7. Parikh, H. *et al.* A resequence analysis of genomic loci on chromosomes 1q32.1, 5p15.33, and 13q22.1 associated with pancreatic cancer risk. *Pancreas* **42**, 209-15 (2013).
8. Liang, J.W. *et al.* Identification of genomic alterations in pancreatic cancer using array-based comparative genomic hybridization. *PLoS One* **9**, e114616 (2014).
9. Gebauer, N. *et al.* Genomic landscape of pancreatic neuroendocrine tumors. *World J Gastroenterol* **20**, 17498-506 (2014).
10. Lubensky, I.A. *et al.* Allelic deletions on chromosome 11q13 in multiple tumors from individual MEN1 patients. *Cancer Res* **56**, 5272-8 (1996).
11. Zhang, J. *et al.* Gene Co-expression analysis predicts genetic aberration loci associated with colon cancer metastasis. *Int J Comput Biol Drug Des* **6**, 60-71 (2013).
12. Wu, C. *et al.* Genome-wide association study of survival in patients with pancreatic adenocarcinoma. *Gut* **63**, 152-60 (2014).
13. Beghelli, S. *et al.* Pancreatic endocrine tumours: evidence for a tumour suppressor pathogenesis and for a tumour suppressor gene on chromosome 17p. *J Pathol* **186**, 41-50 (1998).
14. Bärlund, M. *et al.* Increased copy number at 17q22-q24 by CGH in breast cancer is due to high-level amplification of two separate regions. *Genes Chromosomes Cancer* **20**, 372-6 (1997).
15. Solinas-Toldo, S. *et al.* Mapping of chromosomal imbalances in pancreatic carcinoma by comparative genomic hybridization. *Cancer Res* **56**, 3803-7 (1996).
16. Fong, D. *et al.* Low expression of junctional adhesion molecule A is associated with metastasis and poor survival in pancreatic cancer. *Ann Surg Oncol* **19**, 4330-6 (2012).

17. Uyama, H. *et al.* Hepatoma-derived growth factor is a novel prognostic factor for patients with pancreatic cancer. *Clin Cancer Res* **12**, 6043-8 (2006).
18. Furukawa, T. *et al.* Whole-exome sequencing uncovers frequent GNAS mutations in intraductal papillary mucinous neoplasms of the pancreas. *Sci Rep* **1**, 161 (2011).
19. Chung, F.H., Lee, H.H. & Lee, H.C. ToP: a trend-of-disease-progression procedure works well for identifying cancer genes from multi-state cohort gene expression data for human colorectal cancer. *PLoS One* **8**, e65683 (2013).
20. Ni, S. *et al.* Expression and clinical role of NF45 as a novel cell cycle protein in esophageal squamous cell carcinoma (ESCC). *Tumour Biol* **36**, 747-56 (2015).
21. Shamanna, R.A. *et al.* The NF90/NF45 complex participates in DNA break repair via nonhomologous end joining. *Mol Cell Biol* **31**, 4832-43 (2011).
22. Dupuy, A.J. *et al.* Activation of the Rap1 guanine nucleotide exchange gene, CalDAG-GEF I, in BXH-2 murine myeloid leukemia. *J Biol Chem* **276**, 11804-11 (2001).
23. Yu, H.F. *et al.* High RIN1 expression is associated with poor prognosis in patients with gastric adenocarcinoma. *Tumour Biol* **33**, 1557-63 (2012).
24. Wang, Q. *et al.* Prognostic significance of RIN1 gene expression in human non-small cell lung cancer. *Acta Histochem* **114**, 463-8 (2012).
25. Fernandez-Ranvier, G.G. *et al.* Candidate diagnostic markers and tumor suppressor genes for adrenocortical carcinoma by expression profile of genes on chromosome 11q13. *World J Surg* **32**, 873-81 (2008).
26. Romano, S., D'Angelillo, A. & Romano, M.F. Pleiotropic roles in cancer biology for multifaceted proteins FKBPs. *Biochim Biophys Acta* (2015).
27. Ellsworth, K.A. *et al.* Contribution of FKBP5 genetic variation to gemcitabine treatment and survival in pancreatic adenocarcinoma. *PLoS One* **8**, e70216 (2013).
28. Hackl, C. *et al.* Activating transcription factor-3 (ATF3) functions as a tumor suppressor in colon cancer and is up-regulated upon heat-shock protein 90 (Hsp90) inhibition. *BMC Cancer* **10**, 668 (2010).
29. Frampton, A.E. *et al.* MicroRNAs cooperatively inhibit a network of tumor suppressor genes to promote pancreatic tumor growth and progression. *Gastroenterology* **146**, 268-77.e18 (2014).
30. Lee, C.N. *et al.* RNA interference characterization of proteins discovered by proteomic analysis of pancreatic cancer reveals function in cell growth and survival. *Pancreas* **41**, 84-94 (2012).
31. Buganim, Y. *et al.* p53 Regulates the Ras circuit to inhibit the expression of a cancer-related gene signature by various molecular pathways. *Cancer Res* **70**, 2274-84 (2010).
32. Yoon, K. *et al.* Activation of nuclear TR3 (NR4A1) by a diindolylmethane analog induces apoptosis and proapoptotic genes in pancreatic cancer cells and tumors. *Carcinogenesis* **32**, 836-42 (2011).
33. Zhao, L. *et al.* MicroRNA-383 regulates the apoptosis of tumor cells through targeting Gadd45g. *PLoS One* **9**, e110472 (2014).
34. Liang, C. *et al.* MicroRNA-33a-mediated downregulation of Pim-3 kinase expression renders human pancreatic cancer cells sensitivity to gemcitabine. *Oncotarget* **6**, 14440-55 (2015).
35. Liang, C. *et al.* miR-33a suppresses the nuclear translocation of β-catenin to enhance gemcitabine sensitivity in human pancreatic cancer cells. *Tumour Biol* (2015).
36. Zhang, R. *et al.* Levels of HOXB7 and miR-337 in pancreatic ductal adenocarcinoma patients. *Diagn Pathol* **9**, 61 (2014).
37. Zhang, R. *et al.* miR-337 regulates the proliferation and invasion in pancreatic ductal adenocarcinoma by targeting HOXB7. *Diagn Pathol* **9**, 171 (2014).
38. Barroso-del Jesus, A., Lucena-Aguilar, G. & Menendez, P. The miR-302-367 cluster as a potential stemness regulator in ESCs. *Cell Cycle* **8**, 394-8 (2009).

39. Bai, Z. *et al.* MicroRNA-153 is a prognostic marker and inhibits cell migration and invasion by targeting SNAI1 in human pancreatic ductal adenocarcinoma. *Oncol Rep* **34**, 595-602 (2015).
40. Yang, J. & Zeng, Y. Identification of miRNA-mRNA crosstalk in pancreatic cancer by integrating transcriptome analysis. *Eur Rev Med Pharmacol Sci* **19**, 825-34 (2015).
41. Hamada, S., Masamune, A., Miura, S., Satoh, K. & Shimosegawa, T. MiR-365 induces gemcitabine resistance in pancreatic cancer cells by targeting the adaptor protein SHC1 and pro-apoptotic regulator BAX. *Cell Signal* **26**, 179-85 (2014).
42. Wellner, U. *et al.* The EMT-activator ZEB1 promotes tumorigenicity by repressing stemness-inhibiting microRNAs. *Nat Cell Biol* **11**, 1487-95 (2009).
43. Tréhoux, S. *et al.* Micro-RNAs miR-29a and miR-330-5p function as tumor suppressors by targeting the MUC1 mucin in pancreatic cancer cells. *Biochim Biophys Acta* (2015).
44. Zhou, L. *et al.* MicroRNA-183 is involved in cell proliferation, survival and poor prognosis in pancreatic ductal adenocarcinoma by regulating Bmi-1. *Oncol Rep* **32**, 1734-40 (2014).
45. Yu, J., Li, A., Hong, S.M., Hruban, R.H. & Goggins, M. MicroRNA alterations of pancreatic intraepithelial neoplasias. *Clin Cancer Res* **18**, 981-92 (2012).
46. Nakata, K. *et al.* Micro RNA-373 is down-regulated in pancreatic cancer and inhibits cancer cell invasion. *Ann Surg Oncol* **21 Suppl 4**, S564-74 (2014).
47. Zhang, Y. *et al.* A novel epigenetic CREB-miR-373 axis mediates ZIP4-induced pancreatic cancer growth. *EMBO Mol Med* **5**, 1322-34 (2013).
